# Innate immune-inflammatory signaling milieu in myeloid leukemia and aging-associated clonal hematopoiesis pathologies

**DOI:** 10.3389/fimmu.2025.1660709

**Published:** 2025-10-16

**Authors:** Satyaki Bhowmik, Anwesha Bose, Amitava Sengupta

**Affiliations:** ^1^ Stem Cell & Leukemia lab, CSIR-Indian Institute of Chemical Biology, IICB-Translational Research Unit of Excellence, Kolkata, West Bengal, India; ^2^ Academy of Scientific & Innovative Research (AcSIR), Ghaziabad, Uttar Pradesh, India; ^3^ CSIR-IICB-Cancer Biology & Inflammatory Disorder Division, Kolkata, West Bengal, India

**Keywords:** hematopoiesis, innate immunity, inflammation, acute myeloid leukemia, pattern recognition receptors, mutation, cytokines, interferon signaling

## Abstract

Age-related accumulation of somatic mutations in hematopoietic stem and progenitor cells (HSPCs), causing clonal hematopoiesis (CH), often precedes the development of hematologic malignancies. Chronic inflammation and aberrant cytokine expression that are common in aging, contribute to clonal expansion and genomic instability. Acute myeloid leukemia (AML) is an (epi)genetically and physiologically diverse malignancy, characterized by clonal proliferation and incomplete differentiation of HSPCs. The innate immune system, with pattern recognition receptors (PRRs), plays a pivotal role in maintaining hematopoietic homeostasis. Dysregulated signaling through PRRs disrupts hematopoiesis, fostering malignant cell proliferation. In addition, cytokines and interferons exert multifaceted effects on HSPCs, impacting their proliferation, differentiation, and survival. Therapeutic approaches targeting innate immune pathways, offer promising avenues for treating hematologic malignancies. Understanding the intricate crosstalk between innate immunity and hematopoiesis would provide insights into novel therapeutic strategies for combating hematologic malignancies, offering hope for improved patient outcomes and survival. In this review, we discuss about the malfunctioning innate immune-inflammatory axes in the context of abnormal hematopoiesis and the therapeutic approaches that are utilizing/targeting these pathways with efficacy. This review delves into the derangements of innate immune and inflammatory pathways implicated in the development of AML and myelodysplastic syndromes (MDS), shedding light on the therapeutic strategies targeting these pathways.

## Introduction

The tightly regulated process of hematopoiesis ensures an uninterrupted production of blood cells throughout an individual’s lifespan. However, the hematopoietic system undergoes significant alterations with aging that predispose individuals to cytopenias, clonal hematopoiesis (CH) and hematologic malignancies. A major driver of these age related alterations is the gradual accumulation of mutations in hematopoietic stem and progenitor cells (HSPCs). A subset of these mutations gives a competitive advantage to the mutant clones allowing their disproportionate contribution to circulating blood cells. This condition, known as CH, has been connected to systemic inflammation and an elevated risk of myeloid malignancies. Myelodysplastic syndrome (MDS) and acute myeloid leukemia (AML) are the conditions that best illustrate the malignant manifestation of disturbed hematopoiesis. MDS often develops into secondary AML and is characterized by bone marrow failure, dysplastic hematopoiesis and recurrent somatic mutations. *De novo* AML, on the other hand, develops without a previous history of hematological disease but exhibits clonal dominance of genetically altered HSPCs with impaired differentiation.

Beyond genetic abnormalities, growing evidence highlights that chronic inflammation and innate immune dysregulation play a significant role in shaping the clonal landscape. Inflammatory signals can provide a selection pressure promoting the expansion of preleukemic clones. Thus, innate immune signaling emerges as a key regulator of normal hematopoiesis as well as leukemic transformation. The HSC niche is altered, aberrant myelopoiesis is promoted, clonal progression toward malignancy is accelerated when these pathways are dysregulated. In this review, we provide an overview of how innate immune signaling, aging, and CH interact with hematopoietic abnormalities. We highlight new treatment approaches that target these dysregulated networks and talk about how different immunological mechanisms contribute to the development of MDS and AML.

## Hematopoietic abnormalities

**Figure 1:**
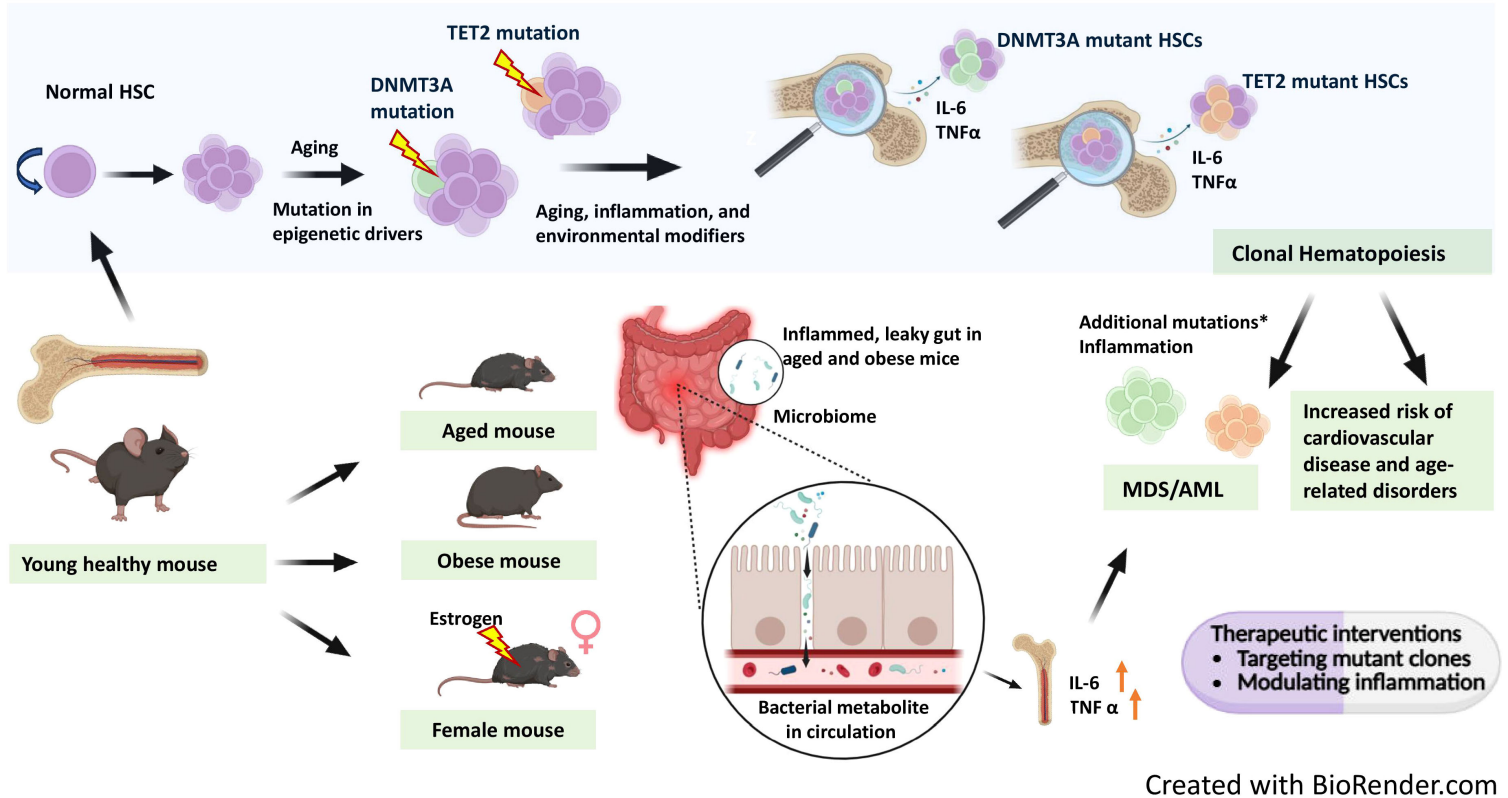
Interplay of age related clonal hematopoiesis with chronic inflammation in leukemia. Aging leads to the spontaneous accumulation of somatic mutations across all cells, including hematopoietic stem cells (HSCs). Many hematological malignancies harbor mutations overlapping with those found in clonal hematopoiesis (CH). CH can also arise as a consequence of chronic inflammation. With aging, mutations in epigenetic drivers like DNMT3A and TET2 accumulate, leading to expansion of mutant HSC clones. DNMT3A mutant clones expand further in female mice under the influence of estrogen. Mouse models of aging and obesity reveal gut inflammation and microbiome dysbiosis, resulting in a leaky gut that allows bacterial metabolites into circulation, further promoting systemic inflammation. Mutant HSCs produce elevated pro-inflammatory cytokines IL-6 and TNF-α, contributing to chronic inflammation. This chronic inflammatory environment, combined with additional mutations in genes involved in key pathways such as RNA splicing, epigenetic modification, cohesin complex function, transcription, DNA damage response, and signal transduction, can drive progression from myelodysplastic syndrome (MDS) to secondary AML. Ultimately, CH and associated inflammation increase the risk of cardiovascular disease and other age-related disorders. Therapeutic interventions targeting mutant hematopoietic clones and inflammatory pathways may help prevent progression from clonal hematopoiesis to myeloid malignancies. The figure is created with BioRender.com.

Aging results in an accumulation of somatic mutations, that develop spontaneously in all the cells present in the body (1, 2). Base substitution mutations occur at an annual rate of 14.2 mutations, which indicate that the adult HSPCs acquire most of the mutations during the post-natal period (3). Some rare mutations grant an advantage to their host HSPCs and their progeny cells (clones) to grow successively and contribute a significant proportion to the adult mature blood cells pool, leading to CH, which is more prevalent with aging (4) (**Figure 1**). Since chronic inflammation is also linked to aging, one possible reason behind CH may be chronic inflammation (5). Most of the hematological malignancies harbor mutations that are common with those found in CH (6) (CH). However, the mutations found in the hematological malignancies may be present in healthy adult population as well (7) and to differentiate the non-malignant CH from the malignant one, a term CHIP (clonal hematopoiesis of indeterminate potential) was coined (8). According to Steensma, D. P. et al., CHIP comprises patients with cytopenia and cancer-associated mutations with no clinically detectable MDS as well as adults with normal peripheral blood counts. Though CHIP itself is not a disease, those who carry CHIP mutations, have been shown to develop MDS/AML at an annual rate of 0.5-1% with a greater probability of disease progression in patients with a higher VAF (9).

When a comprehensive diagnosis including a bone marrow examination is inconclusive in a patient appearing with one or more peripheral blood cytopenias, the term ‘idiopathic cytopenia of undetermined significance’ (ICUS) is used in general (10, 11). Peripheral blood cytopenias present a wide range of differential diagnoses, encompassing autoimmune diseases, inflammatory conditions, and various bone marrow disorders such as myelodysplastic syndromes (MDS), aplastic anemia, infiltrative processes, and toxic damage. Normal bone marrow activity can also be suppressed by some pharmaceuticals, as well as dietary inadequacies (12). Although a universally accepted definition of ICUS has not yet been established, the following diagnostic standards have been put forth: (I) at least four months of cytopenia, (II) failure to meet the basic diagnostic requirements for MDS, (III) exclusion of all other causes of cytopenia, and the absence of any genetic abnormalities in myeloid cells, such as mutations, karyotype abnormalities, or flow cytometry aberrations (13).

Patients with cytopenias who do not meet the diagnostic criteria for MDS (having < 10% dysplastic cells inside the bone marrow) or other hematologic illnesses and possess somatic mutations at a variant allele frequency (VAF) of ≥ 2% in genes linked to myeloid malignancies are referred to as having clonal cytopenia of unknown significance (CCUS) (14). The major differences between CHIP and CCUS are, clonal size is often greater in CCUS (VAF of 38% vs. 9% for CHIP), genes linked to a poor prognosis, such ASXL1, RUNX1, and TP53, are more frequently affected, and several genes are impacted in > 64% of CCUS patients compared to 8% of CHIP events (15–17). The comparison among these abnormalities has been discussed in **Table 1**.

**Table 1:** Comparison of CHIP, ICUS and CCUS.

Feature	Clonal hematopoiesis of indeterminate potential (CHIP)	Idiopathic cytopenia of undetermined significance (ICUS)	Clonal cytopenia of undetermined significance (CCUS)
Definition	Presence of somatic mutations in hematopoietic cells without cytopenia or clinically detectable hematologic malignancy	Persistent cytopenia without detectable cause and clonal mutations	Persistent cytopenia with identifiable clonal mutations but without clinically detectable MDS or other hematologic malignancy
Cytopenia	Absent	Present	Present
Somatic mutations	Present	Absent	Present
Bone marrow findings	Normal or age related changes; no dysplasia	No significant dysplasia	May show mild abnormalities but not detectable MDS
Risk of Progression	Increased risk of MDS/AML	Low risk; may progress if clonal evolution occurs	Significant risk of progression to MDS/AML
Diagnosis Requires	NGS to detect clonal mutations	Persistent cytopenia with exclusion of secondary causes	Both cytopenia and NGS but without clinically detectable MDS

MDS is a collective term for describing the hematopoietic stem cell abnormalities in elderly patients. These disorders in common result in dysplasia of bone marrow precursors and peripheral cytopenia along with recurrent somatic gene mutations and chromosomal abnormalities (18). The most prevalent clinical symptom in MDS patients is moderate anaemia, although full myeloid bone marrow failure can also occur which frequently results in bleeding or infection-related mortality (19). Chronic inflammation encourages genomic instability through DNA mutations and epigenetic alterations, prevents tumour immune surveillance, and is predisposed to clonal evolution, all of which favour the development of cancer. As a result, the presence of MDS as well as ongoing chronic inflammation in the BM may eventually encourage the growth of leukemia. MDS patients may progress to secondary Acute myeloid leukemia (AML) that can be diagnosed by increased blast count in the bone-marrow and characterised by difference in proliferation and cell death (20). MDS and secondary AML cases share mutations in genes implicated in at least six key pathways (20).

However, in most of the cases reported, AML arises *de-novo* in healthy individuals with no previous report of hematological malignancies (21). AML can be characterized by uncontrolled clonal proliferation of immature hematopoietic precursors and incompletely differentiated myeloid blasts leading to impaired hematopoiesis in adults (22). The clonal proliferation and poor differentiation of mutant HSPCs define acute myeloid leukemia (AML), a genetically and physiologically diverse malignancy (23). Chimeric proteins are formed as a result of well-studied chromosomal translocations, which change the normal differentiation and maturation processes of myeloid precursor cells. However, in majority of AML patients, no large chromosomal abnormality is found (24), rather mutations are the key role players in those cases (25). Animal model studies have allowed to classify these mutations into different classes (26) like class I, which includes mutations in FLT3 (ITD and TKD), K/NRAS, TP53, c-KIT and class II, including NPM1 and CEBPA mutations (21). A third class of mutations include those in the epigenetic regulators like DNMT3A, TET2, IDH-1 and IDH-2 (21). Even before patients are diagnosed with AML, they may carry more mutations in some of these genes (DNMT3A, TET2, TP53, SRSF2, IDH2, SF3B1, JAK2, ASXL1), compared to control individuals and having any of these mutations at baseline assessment can be associated with a statistically significant increased risk of developing AML (27). Individuals, who have an autoimmune or chronic immune-stimulated condition, are more likely to acquire AML (28). This connection suggests that dysregulated cytokine expression, a common feature of auto-inflammatory disorders and chronic inflammation, may also encourage the growth of hematological malignancies. Although the role of genetic mutations and epigenetic modifications in the development of these malignancies have been well investigated, recent studies have found dysregulated immune and inflammatory axis to be one of the major players in the pathogenesis of AML.

## Components of innate Immune system and their role in hematopoiesis

Germ line encoded Pattern Recognition Receptors (PRRs), present on the plasma membrane as well as within the cytoplasmic compartment of cellular components of the innate immune system for scanning the extra- and intra-cellular environments, have broad specificity to identify Pathogen Associated Molecular Patterns (PAMPs) and Damage Associated Molecular Patterns (DAMPs), which share a more or less similar structural pattern (29). This is evident from the recent studies that, innate immune signals and pathways that are specific to the activation of innate immune cells have a major role to play in normal, healthy HSPCs to regulate hematopoiesis.

Toll-like receptors (TLRs) are the first discovered PRRs, followed by the discovery of other PRR families and identification and characterization of their respective ligands that can initiate the immune signaling upon ligation. The TLRs play an important role in the innate immune system and can be present on the surface of immune as well as non-immune cells (endothelial and mesenchymal stem cells) as homodimers or heterodimers (30). TLR4 activation and induction of nuclear factor (NF)-κB has been shown to be important for subsequent activation of adaptive immune signaling (31). NF-κB plays a key role in inflammation and stress responses and is a key mediator of cell survival (32). Presence of NF-κB is evidently essential for proper functioning of HSCs including HSC proliferation, differentiation and self-renewal (33). Abnormal TLR signalling may lead to malfunctioning of haematopoiesis or haematopoietic malignancy (34, 35). On the contrary, absence of TLR signalling leads to lesser HSC repopulating defects (36).

An intracellular PAMP-sensing NOD-like receptor (NLR) family of receptors reside in the cytoplasm and is known to exert initial response against injury and stress (37). The members (NLRP1, NLRP3, NLRC4) are known to assemble canonical inflammasomes that are complemented by non-canonical pathways to activate different caspases in animal cells via well characterised mechanisms (37). Nucleotide-binding oligomerization domain-containing protein 2 (NOD2) resembles the membrane-bound TLRs. NOD2 detects pathogen-associated motifs and triggers inflammatory signaling cascades that activate NF-κB (38). Ongoing studies have identified inflammasomes to be role players in the field of promoting human HSPC production. The Nlrp3 inflammasome can detect the influx of glucose and/or amino acids into cells, which causes adult human lymphocytic cells to proliferate and differentiate (39).

Interferons were primarily reported as soluble factors that possess antiviral and growth inhibitory activities (40). Interferons are mainly classified into two types: type I and type II. Type I interferons include IFN-α, IFN-β, IFN-ϵ, IFN-κ, IFN-ω, IFN-δ, and IFN-τ whereas, type II interferons include only IFN-γ (41). Following recognition of pathogen-associated molecular patterns (PAMPs), such as foreign or self-nucleic acids, type I IFNs are produced by the majority of cells (42). IFN signaling is initiated by the ligand-receptor interaction followed by the structural rearrangements of the ligand and dimerization of the receptor subunits and subsequent activation of the receptor associated JAKs leading to direct or indirect regulation of interferon stimulated genes (ISGs) and the downstream pathways (43). These ISGs can be engaged in a variety of activities, including the regulation of the immune system and cell death. IFN-α connects the innate and adaptive immune systems by promoting T cell proliferation and survival as well as NK cell cytotoxicity, in addition to having effects directly on tumour cells (44). IFN-α has been shown to possess the ability to remove the dormancy of HSCs and activate them *in vivo* (45). In contrast to this, chronic exposure to type I IFN takes the HSCs back to quiescence and saves the HSC pool from being exhausted (46).

The complement system was defined on the basis of its capacity to complement the immune responses mediated by cells and antibodies. Upon recognition of PAMPs and/or DAMPs, the complement effector molecules, which are in their precursor state and circulate in the serum and interstitial fluids, get quickly activated in a proteolytic and cascade-like way (47) The components of the complement system can also be produced and expressed locally by different immune and non-immune cell types (48–50). The observation that various cell types appear to contain a diverse range of complement components, receptors and regulators in a manner similar to the inflammasome, the term ‘complosome’ was coined to describe the intracellular complement system (51). The complosome meticulously regulates the production of intracellular reactive oxygen species (ROS) via both mitochondrial and cytosolic signaling pathways, thereby initiating stress-adaptive responses that enhance mitochondrial functionality and improve the overall fitness of HSPCs (52). These components have been extensively reviewed in several articles, thus, we are not citing them and their original references because of space constraints.

## Malfunctioning of innate immune and inflammatory pathways in myeloid malignancies

### Inflammasomes

The NLRP3 inflammasome is the subtype of inflammasome that has the greatest characterization and is implicated in autoimmune homeostasis as well as the pathophysiology of autoimmune and inflammatory disorders. The DAMP proteins S100A8 and S100A9, which are considerably elevated in MDS patient blood plasma, play a key function as activators of the Nlrp3 inflammasome in the pathogenesis of MDS (53). In response to elevated amounts of extracellular DAMPs, activation of the Nlrp3 inflammasome expands BM myeloid-derived suppressor cells, intensifies BM inflammation, damages hematopoietic cells, causes chromosomal abnormalities, eventually inducing pyroptosis (54). Nlrp3 inflammasome activated Caspase-1 is needed for pyroptosis, which subsequently causes cell enlargement, plasma membrane rupture, and the enormous release of intracellular DAMPs (extra cellular ATP, HMGB1, DNA, and ASC oligomers) as well as IL-1 and IL-18, which together recruit additional immune cells in the extracellular space and initiate the inflammatory cascade in the BM microenvironment (55). Nlrp3 inflammasome undoubtedly contributes to the development and progression of myeloproliferative neoplasm, but further in-depth research is required to understand how it affects this disease. Recent research indicates that, leukemic cells’ migratory and lethal dissemination are significantly influenced by the Nlrp3 inflammasome (56).

### Cytokines

Cellular Proliferation, survival and therapy resistance in acute myeloid leukemia (AML) and other hematological cancers are significantly influenced by cytokines, their crucial functions and intricate interplay, in the setting of inflammation. The expression and levels of a number of cytokines (TNF-α, IFN-γ, TGF-β, IL-6 and IL-8) are found to be increased in case of MDS and signify abnormal inflammatory signaling and hematopoietic differentiation (57). Studies have found that individuals, who have chronic inflammation in their system, are more likely to acquire AML (28). An *ex vivo* cell viability screen in which primary AML samples were cultured in the presence of 94 distinct cytokines, to extensively analyse the impact of cytokines in modifying AML cell survival, identified IL-1β, exerting the most robust effects including increased cell growth and survival in almost 70% of 60 primary AML samples, irrespective of the mutational status (58). IL-1β treatment to AML cells increases the production of other cytokines, known to facilitate AML proliferation (59, 60). IL-6 plays important roles within the network of cytokines involved in the formation of leukemic blasts (61). In AML patients, plasma levels of IL-10 are notably elevated and are associated with elevated levels of IL-6 (62). By suppressing pro-leukemic cytokines at the transcriptional or post-transcriptional level, IL-10 may act to prevent the proliferation of AML cells (63). Jaiswal et al. have shown that macrophages from *Tet2^−^* ^/^ *^−^* bone marrow produce increased amounts of CXCL1, CXCL2 and CXCL3 (64). In individuals with Tet2 mutated CHIP, they have found increased IL-8 in serum. *Tet2*-deficient mice have increased IL-1β and inflammasome activation (65). Chronic inflammation and immunologic changes in bone marrow niche microenvironment are the effects of Tet2 deficiency (66). Yeaton et al. have demonstrated that disease development in aged animals is associated with an augmented inflammatory response and the establishment of an abnormal inflammatory monocytic cell population using single-cell transcriptome profiling of the bone marrow (67). In individuals with AML, the gene profile that characterises this inflammatory population is linked to a poor prognosis. S100A8 and S100A9 are damage associated molecular patterns (DAMPs), that are expressed in a variety of cells like endothelial cells, macrophages, osteoclasts, keratinocytes and dendritic cells upon inflammatory challenges (68) and can act as endogenous ligands for TLR4, resulting in dysregulated hematopoiesis in MDS by affecting HSPCs directly or indirectly (69). It has been shown that the S100A8 and S100A9 transcripts are overexpressed in the M4 and M5 AML compared to that in M0 and M1 (70). S100A8 and S100A9 appear to influence immunological check points to control leukemic proliferation (71).

### Interferons

A study comparing the bone marrow-derived mesenchymal stem cells from 7 MDS patients found an upregulation of IFN-α/β signaling and ISG15 expression (72). MDS patients have an overexpression of IFN-γ in the bone marrow cells (73). In AML cells, chromosomal instability has been shown to result in micronuclei, subsequent DNA damage, and interferon signaling (74). During chronic infection, impairment of an IFN-γ mediated transcriptional network causes proliferation of Dnmt3a-loss of function HSC clones in a mouse model by decreasing stress induced apoptosis and inducing defective differentiation (75). IFN-γ secreted by T cells in the bone marrow microenvironment induces the expressions of PD1 on T cells and PDL1 on tumor cells resulting in the immune escape of the malignant cells (76). In the presence of recombinant interferons, colony forming ability of blast progenitors from peripheral blood isolated from AML patients was found to be suppressed relative to the control cultures (77). IFN-γ has been shown to have a suppressive effect on clonal proliferation of leukemic blasts and induce differentiation of the blasts isolated from patients with AML and CML (78). HSC function can be compromised upon chronic exposure to inflammation/inflammatory challenges (46, 79, 80). Several interferon-stimulated genes, including STAT1, IRF9, IFIT1, IFIT3, IFITM1 and IFI44L, were found to be upregulated in the CD34^+^ cells of MDS patients (81). Through interactions between the leukemic and mesenchymal stem cells, a subset of AML patients with high IFN-γ levels result in expansion of regulatory T cells (82).

### Pattern recognition receptors

A number of TLRs like TLR2, TLR4 AND TLR6 have increased expression on the bone marrow CD34^+^ cells in MDS patients (83). Increased TLR2 and TLR4 levels in MDS patients are correlated with increased rates of apoptosis (84, 85). Despite increased expression with other TLRs in bone marrow cells during initial stage of MDS, TLR9 expression exhibits a significant downregulation when MDS gets transformed into AML (86). TLR2 and TLR4 act as receptors for HMGB1 (87) and knockdown of these receptors exhibited reversal of NLRP3 induction resulting from HMGB1 treatment (88). NF-κB activity was found to be increasing with the progression of MDS and was highest in the later stages of the disease (89), especially in elderly patients above the age of 75 years (90). Primary AML CD34^+^ cells exhibit easily observable NF-κB activity, in contrast to human CD34^+^ progenitor cells, which do not express NF-κB in the absence of stimulation (91). When NF-κB was inhibited in AML cell lines as well as in primary blasts isolated from AML and MDS patients, nutrient depletion driven apoptosis could be induced (92). During induction chemotherapy in AML patients, polymorphisms in TLR2 and TLR4 may result in infectious complications (93). CLL-1, also known as C‐type lectin domain family 12, member A (CLEC12A), has been identified as a marker for leukemic stem cells as it is present on the surface of CD34^+^CD38^-^ cells in AML patients but can not be found on CD34^+^CD38 cells from healthy individuals (94, 95). The role of the cytokines and the PRRs have been discussed in **Table 2**.

**Table 2:** Pathophysiological role of innate immune signaling components in leukemia.

Molecule	Normal function	Expression	Role in leukemia
TLR-4	PRR, binds to LPS	Increased in MDS bone marrow, polymorphism	Increased apoptosis, Infectious complications
TLR-1/2	PRR, binds to lipoproteins	Increased In MDS bone marrow, polymorphism	Increased apoptosis Infectious complications
NF-κB	Transcription factor, modulates cellular response to stress and inflammation	Increased in later stages of MDS	Apoptosis driven by nutrient depletion
NLRP3	PRR, assembles inflammasomes	Increased	Contributes to MPN progression
IL-1, IL-6, IL-7	Cytokine, activates myelopoiesis during inflammation, maintains peripheral T cell counts	Increased in MDS	Increases cytokine production facilitating AML proliferation, facilitates leukemic blast formation
Type I Interferon	Cytokine, regulates immune system and cell death via ISGs, removes HSC quiescence	Increased	Increased ISG expression
Type II Interferon	Cytokine, regulates HSC quiescence and differentiation	Increased in MDS bone marrow cells, Impaired transcriptional network	Provides advantage to *Dnmt3a*-mutant HSC clones, results in immune escape of malignant cells, facilitates expansion of T_reg_ cells
S100A8 & S100A9	Cytokines, act as DAMPs	Elevated in MDS blood plasma, overexpressed in M4 and M5 AML	Activates NLRP3 inflammasome and causes pyroptosis leading to initiation of inflammatory cascade, dysregulation of hematopoiesis in MDS

### Complosome

Various leukemic cell lines like HEL, K562, HL-60, HG-1a show mRNA expression for C3, C5, C3aR and C5aR1 (96). The potential function of the complosome in modulating the biology of these cells was found by deletion of the expression of C3 and C5 using CRISPR-Cas9, which hampered the migration of leukemic cells toward SDF-1 and eATP. C3 and C5 mRNA were also found to be significantly upregulated in AML blasts isolated from peripheral blood.

### Immune signaling genes

Several cytokines, including interleukins and interferons have the ability to activate Janus Kinase 2 (JAK2), which regulates hematopoiesis. By fostering STAT phosphorylation, activated JAK2 primarily communicates with the STAT proteins. The transcription of downstream target genes is facilitated by subsequent dimerization and nuclear translocation of STAT. Numerous interleukins, such as IL-6, influence immune-inflammatory response, and immune cell development via signaling through the JAK-STAT pathway (97). JAK2 V617F is one of the most identified CH mutations, based on which different genetic murine models have been developed. Most of these mice exhibit MPN characteristics consistently (98, 99). Although it is not that clear how the same JAK2 mutation gives rise to different MPN subtypes, probably it is related to the involvement of different STATs in different subtypes (100).

## Role of the bone marrow microenvironment/niche in CH progression

CH has been found to be strongly associated with infection, age-associated cytokine overproduction and other external inflammatory stimuli in the bone marrow microenvironment (101, 102). Pro-inflammatory cytokines released by immune cells to clear-off pathogens can stimulate the production of intracellular reactive oxygen and nitrogen species in adjacent cells within the microenvironment, setting a stage for somatic mutations in HSCs (103). These mutations may result in skewed differentiation and myeloid restricted progenitors. These devoted progenitors may have increased inflammatory signature that maintain a highly inflamed microenvironment for CH. As the inflammatory bone marrow ecosystem may include selection forces that promote the growth of CH clones, HSPCs with CH mutations may survive and be enriched throughout this process (104). For instance, *Tet2*-deficient HSPCs show increased expression of an anti-apoptotic long non-coding RNA as a result of IL-6-induced hyperactivation of the Shp2-Stat3 signaling cascade. *Tet2* deficient HSCs are therefore resistant to apoptosis because of the inflammatory cytokine (105). Through impaired RIPK1-RIPK3-MLKL-mediated necroptosis signaling, *Dnmt3a*-mutated bone marrow cells also exhibit enhanced reconstitution potential in an aged bone marrow milieu in response to inflammatory insults (TNF-α) (106, 107). Although the underlying processes are unknown, the HSPCs with an *Asxl1* mutation showed an anti-inflammatory gene expression profile (108). Regardless, one recent study has underscored telomere attrition as an instrument for clonal selection in aging hematopoiesis and leukemogenesis (109). Together, HSPCs with CH mutations gain clonal dominance over their normal counterparts by upregulating anti-inflammatory or anti-apoptotic genes, which increase their fitness in an inflammatory environment, and confer resistance to apoptosis.

Recent findings have shown that a few important components of the osteo-hematopoietic niche, such as mesenchymal stromal cells, endothelial cells, osteolineage cells, and the regulatory signals they transmit such as CXCL12, SCF, and osteopontin, gradually deteriorate with age. This degradation promotes the selection of mutant clones while also making the environment less favorable to normal hematopoietic stem cells (HSCs). Additionally, inflammatory cytokines (such as IL-1β, IL-6, and TNF-α) secreted by mutant HSPCs and their descendants actively remodel the bone marrow niche, reinforcing pro-inflammatory signaling loops and causing additional niche dysfunction (110) (**Figure 1**). Thus, a bidirectional interaction between mutant clones and their aging microenvironment shapes CH development, which is not just controlled by HSPC-intrinsic mutations. The influence of the aging microenvironment on clonal dynamics was illustrated in an elegant study employing *DNMT3A R878H*-mutant models (111). Transplantation studies performed into both young and elderly recipients demonstrated that *DNMT3A*-mutant HSCs acquire a competitive edge in aged bone marrow settings, which is mediated by increased TNF-α signaling. By influencing lineage bias toward myeloid vs lymphoid output, TNFR2 uncouples clone size from differentiation trajectory, whereas TNFR1 stimulates mutant clone proliferation. In addition to intrinsic signals from the stromal, endothelial and immune cells, microbial agents have been found to be important regulators of the bone marrow microenvironment in CH. Microbial components and PAMPs alter HSPC function and stimulate pre-leukemic myeloproliferation in *Tet2*-deficient models. For example, in *Tet2*-deficient mice, myeloproliferation can be induced by commensal microbiota-derived chronic inflammatory signaling, mainly through the IL-6 and IL-1β pathways (112). According to Yokomizo-Nakano et al., *Tet2* loss after sequential exposure to microbial products enhances the signs of MDS, indicating that microbial signals could modify hematological niche for the growth of mutant clones in addition to their clonal expansion (113). Long-term IFN-γ exposure or chronic mycobacterial infection promotes the clonal proliferation of *Dnmt3a*-mutant HSCs through impaired differentiation and epigenetic reprogramming (106). Additionally, microbial translocation and dysbiosis offer a steady supply of PAMPs and metabolites that may affect HSPC activation and lineage output by altering systemic cytokine profiles (114). Overall, aging-associated intestinal alterations can cause systemic dissemination of microbial metabolites for pre-leukemic cell expansion. In this regard one recent elegant study has indicated that microbial metabolite [ADP-D-glycero-β-D-manno-heptose (ADP-heptose)], drives CH via ALPK1, suggesting that the ADP-heptose-ALPK1 axis is a promising therapeutic target to prevent progression of CH to overt leukemia and immune-related conditions (115). These results demonstrate that the bone marrow microenvironment actively influences the fitness and evolution of CH clones. Furthermore, they emphasize the idea that inflammatory remodeling of the hematopoietic niche with aging produces a milieu that is favorable for CH and may hasten the progression of AML/MDS.

## Therapeutic approaches targeting innate immune pathways involved in CH

Poor progress has been achieved in prolonging the survival of high-risk people with AML. Although the use of targeted treatments in combination with chemotherapy has enhanced the chances of complete remission, recurrence rates have not changed much and remain the leading cause of treatment failure in the post-consolidation or post-transplantation setting. As a result, there is an increasing need to develop efficient and secure post-remission therapy strategies that may improve disease free survival and, as a result, overall survival.

IFN has the ability to stimulate apoptosis, inhibit cytokines that promote cell growth and proliferation, and increase the immunogenicity of AML cells (116). These immunomodulatory and anti-proliferative effects emphasise IFNs as a possible therapy for myeloid malignancies (117), especially in cases where the patients are tolerant to the conventional therapies. Several studies have shown that Interferon’s anti-leukemic efficacy is due to both direct action on leukemic blasts and indirect action through immune activation. IFN-α induces latent HSCs to enter the cell cycle (118). This suggests that administering IFN to AML patients can cause quiescent LSCs to multiply and become susceptible to conventional treatment. Minimal residual disease positive AML patients with t(8;21) mutations have reported a better 2-year overall survival upon IFN-α treatment after allogenic hematopoietic stem cell transplantation (119). It has been demonstrated that IFN- α maintenance therapy can lower the probability of relapse in AML patients with favourable risk (120). An ongoing phase I/II trial has shown that pegylated IFN-α did not significantly alter toxicity or acute graft-versus-host-disease (GVHD) risk when administered prophylactically after myeloablative conditioning in an AML cohort at high risk for relapse and resulted in relatively low rates of relapse suggesting a robust graft versus leukemia (GVL) response (121). Stimulating endogenous IFN synthesis, for example by turning on the cyclic GMP-AMP synthase (cGAS)-stimulator of interferon genes (STING) pathway, is one method of exploiting interferon’s anti-cancer capabilities (122). cGAS/STING-dependent anti-leukemic action was demonstrated by the STING activator DMXAA in a mouse model of AML either by significantly prolonging the survival, or in some cases, curing the mice (123).

The innate immune system includes NK cells, which are able to detect the lack of certain proteins that may be downregulated on malignant cells, such as HLA proteins. They can also obliterate tumour cells right away. *In vitro* transformation of primary human NK cells into memory-like NK cells results in the production of IFN-γ and the manifestation of anti-leukemic characteristics (124). The first in human phase I study included three patients with relapsed/refractory AML received treatment with anti-CD33 CAR NK-92, but resulted in no durable response (125). Engineered CAR-NK cell treatments are becoming a popular new kind of therapy (126). TLR3,7,8, and 9 have been the focus of most of the clinical trials employing TLR agonists to treat hematological malignancies (127). A TLR8 ligand R848 has been shown to induce differentiation and stop proliferation of AML cells (128). It has been shown that two SNPs in the human *TLR9* gene, T1486C and T1237C, downregulate the expression of TLR9 mRNA resulting in an improved outcome of graft transplant in AML patients (129). As the malfunctioning inflammatory pathways are one of the key responsible factors for maintaining the leukemic state of the immune cells, as a cell-intrinsic, self-directed immunotherapy these pathways may be applied. Ellegast et al. has shown using different genetic and protein degradation techniques *in vitro* and genetically *in vivo* to demonstrate AML cells’ reliance on IRF2BP2, which is shown to suppress IL-1β/TNFα signaling via NF-κB, and IRF2BP2 disruption causes an acute inflammatory state that culminates in AML cell death (130). In the mouse model of AML, therapy with an anti-S100A8 antibody led to a comparable effect on AML cell differentiation as treatment with recombinant S100A9 protein, both of which increased survival (70). S100A9 transgenic (S100A9Tg) mice and BM-MNC treated with S100A9 activate the programmed death 1 (PD-1) and programmed death-ligand 1 (PD-L1). MDS BM-MNC treated with recombinant PD-L1 experienced cell death, and in turn, PD-1/PD-L1 inhibition increased colony-forming ability in MDS patient BM-MNC and restored efficient hematopoiesis (71). Buteyn et al. have shown that a combination of synthetic NOD2 ligand and IFN- γ produced an inflammatory cytokine profile and activated NK cells in AML blasts which resulted in significantly increased mature CD27^-^ CD11b^+^ NK cells in a mouse AML model, as well as significantly decreased disease burden and prolonged survival (131).

Even though CH is mostly linked to negative clinical outcomes, such as an elevated risk of systemic inflammation and hematologic malignancies, it is increasingly becoming evident that not all mutations linked to CH have harmful effects. The immune system may benefit functionally from certain mutations in some cases. CH mutations can increase the effectiveness of chimeric antigen receptor (CAR) T-cell treatment by improving the anticancer activity and T-cell persistence (132). Evidence from models of neurodegenerative diseases indicates that CH can stimulate positive immune responses in addition to cancer immunotherapy; CHIP carriers showed improved outcomes in Alzheimer’s disease and increased microglial activation, suggesting a possible protective role in neuroinflammation (133, 134). Spetzler et al. have identified mutations linked to CHIP in solid tumors that affect tumor-infiltrating lymphocytes (TILs) (135). This finding suggests that hematopoietic clones with CH mutations may directly impact the tumor-immune interface, possibly impacting immunotherapeutic response as well as immune surveillance. These results imply that CH should be considered a physiologically diverse state where the functional effects of mutations are extremely context-dependent, rather than just a pre-malignant or pathogenic condition.

## Conclusion & future perspectives

Overall, the members of the innate immune system, like PRRs as well as cytokines and chemokines, have crucial roles in the regulation of the hematopoietic system. Malfunctioning of the components, as well as abnormal activation of the signaling pathways involved in the intricate interplay between these two systems may lead to genomic instability, compromised hematopoiesis and hematopoietic abnormality/malignancy like MDS and AML. The fact that CH can have context-dependent consequences, such as potentiating therapeutic responses or providing immunological benefits, further demonstrates that the biology of this process is more complex than previously appreciated. Current therapeutic approaches have shown exceptional efficacy and may prove themselves to be more effective in the treatment of AML in future. However, finding safe, well-tolerated post-remission techniques that can lower the relapse rate is a continuous problem in the treatment of AML. In cases of complete remission post stem cell transplantation, in specific subsets of AML patients maintenance therapy can be an important player (116). There is a dire need to sustain morphological and/or molecular complete remission in older patients, who have poor biological features at baseline.

Even with the significant advancements made in the field of CH, there are still many unanswered questions and unexplored areas. For example, early-life HSCs may be inherently less likely to acquire or spread somatic mutations, as shown by the low frequency of CH in children. Long-term effects on clonal evolution may arise from prenatal exposure to inflammation caused by environmental pollutants, which could still provide a mutagenic environment *in utero*. Early-life immune surveillance, in comparison to later stages of life, may provide better immunity against the outspread of mutant clones. However, as people age, the bone marrow microenvironment may get reprogrammed conducive to the proliferation and selection of pre-cancerous clones, which might lead to a higher frequency of CH among elderly individuals. Further influencing the process of CH development is the probable difference in the microbial components between childhood and adulthood that interact with the hematopoietic and immune systems. Future investigations addressing these aspects will improve our overall understanding of the processes in which immune competence, environmental exposure, stem cell physiology, ontogenty and microenvironmental signals interact to influence clonal development throughout life. Comprehensive systems biology understanding of immune signaling in the context of physiological aging associated clonal hematopoiesis, by leveraging genetic and meta-analysis coupled with cutting edge single-cell mutlimodal studies, will provide important insights in innate immune signaling and blood cell homeostasis.
